# A mixed-methods study of a “Humanities in Course” model: improving caring abilities, climate, and satisfaction in pediatric nursing education

**DOI:** 10.1186/s12909-026-09809-0

**Published:** 2026-06-29

**Authors:** Huang Chen, Shumaila Batool, Longhui Lai, Jieqiong Zhang, Leshan Zhou, Juan Wang, Yingru Wang

**Affiliations:** 1 Teaching and Research Section of Clinical Nursing, Xiangya Hospital of Central South University, Changsha, China; 2 Department of Nursing Science, University of Turku, Turku, Finland; 3 Department of Urology, The Second Clinical Medical School, Lanzhou University Second Hospital, Gansu Province Clinical Research Center for Urinary System Disease, Lanzhou, China; 4 Department of Nursing, Lanzhou University Second Hospital, Lanzhou, China; 5 Xiangya Nursing School, Central South University, Changsha, China; 6 School of Nursing, Lanzhou University, Lanzhou, China

**Keywords:** Caring abilities, Humanistic care, Mixed methods, Nursing education, Pediatric nursing, Student satisfaction

## Abstract

**Background:**

Pediatric nursing requires exceptional humanistic care. However, humanistic education is often delivered separately from professional nursing courses, creating a gap between caring principles and clinical practice.

**Aim:**

This mixed-methods study evaluated the effectiveness of the “Humanities in Course” model in enhancing nursing students’ humanistic caring abilities and improving the caring climate in pediatric nursing education.

**Methods:**

A concurrent mixed-methods approach was adopted. Using a non-probability whole-population sampling method, all third-year undergraduate nursing students enrolled in the Pediatric Nursing course during the 2023 academic year were invited to participate. Seventy-five students who completed the course and provided both pre- and post-course data were included in the final analysis. Quantitative data were collected before and after the course using the Caring Ability Inventory, Peer Group Caring Interaction Scale, and Organizational Climate for Caring Questionnaire, and were analyzed using descriptive statistics and paired-sample t-tests. Students’ perceptions of the course were evaluated after the intervention using a self-developed Teaching Effectiveness Evaluation Questionnaire. Qualitative data were collected from students’ narrative diaries written after the RealCare Baby^®^ 3 experiential learning activity and analyzed using Colaizzi’s seven-step method with NVivo 12.

**Results:**

Students’ total caring ability scores increased significantly from 188.11 ± 18.69 before the course to 203.57 ± 16.26 after the course (*p* < 0.001). Significant improvements were also observed in the total scores of the Peer Group Caring Interaction Scale and the Organizational Climate for Caring Questionnaire after the intervention. The teaching effectiveness evaluation showed that most students perceived the course positively, with 94.7% reporting curricular attractiveness, 96.0% reporting improved analytical problem-solving ability, and 98.7% reporting enhanced patience when interacting with patients. Qualitative analysis of narrative diaries identified five themes: gratitude to parents, professional quality development, bioethics, operational learning, and course experience.

**Conclusion:**

The “Humanities in Course” model may be a feasible approach for embedding humanistic education into pediatric nursing education. It was associated with improvements in students’ overall humanistic caring ability, peer caring interaction, and perceived caring climate, while qualitative findings indicated perceived development in professional quality, bioethical awareness, operational learning, and reflective understanding of pediatric caring. Future controlled, multi-center, and longitudinal studies with objective and multi-source outcome measures are needed to confirm the effectiveness, sustainability, and transferability of this model.

**Supplementary Information:**

The online version contains supplementary material available at https://doi.org/10.1186/s12909-026-09809-0.

## Background

Humanistic care is a fundamental aspect of nursing’s professional culture. It involves providing patients with both professional skills and emotional support, grounded in nurses’ professional knowledge and caring values [1, 2]. With the development of holistic and person-centered nursing models, human dignity, respect for life, and emotional support have become increasingly important in nursing practice [3]. Humanistic caring ability reflects nurses’ capacity to proactively care for, empathize with, respect, and serve patients, and it is regarded as an essential professional competence in nursing [4–6]. Internationally, humanistic and person-centered approaches have also received increasing attention in nursing education. Nursing students are expected not only to acquire technical competence but also to develop empathy, ethical sensitivity, communication skills, reflective capacity, and the ability to respond to patients’ psychological and relational needs. Recent evidence suggests that narrative medicine and narrative writing can enhance nursing students’ professionalism, empathy, and humanistic caring ability [7], while simulation-based learning, ethics education, and experiential learning can support students’ caring attitudes, professional identity, and readiness for clinical practice [8–11]. However, nursing students still report unmet needs related to humanistic course provision, teaching methods, and humanistic learning environments [12].

Humanistic pedagogical models are particularly necessary in pediatric nursing education. Children are physically, cognitively, and emotionally vulnerable, and pediatric nursing requires timely, individualized, and developmentally appropriate care [13]. Children’s illness experiences are closely connected with their families’ anxiety, expectations, and decision-making, making family-centered communication an essential component of pediatric care. Compared with adult patients, children may have limited ability to express pain, fear, or discomfort, and they often depend on nurses and parents to interpret and respond to their needs. Simulation-based pediatric education has been reported to enhance students’ practical competence, empathy, and readiness for pediatric care [11]. Recent scoping review evidence on pediatric patient- and family-centered care has highlighted the need for educational programs that strengthen communication, relational competencies, and attitudes toward children and families [14]. A further scoping review showed that pediatric simulation in undergraduate nursing education can improve students’ self-confidence, knowledge, clinical judgment, skills, and satisfaction [15]. Similarly, infant-care simulation can help learners understand caregiving responsibility and parental perspectives through experiential learning [16]. Without systematic humanistic education, nursing students may focus predominantly on technical tasks while overlooking children’s psychological comfort, family communication, relational needs, and ethical concerns. Such omission may lead to insufficient empathy, ineffective communication with children and parents, inadequate emotional support, increased distress during care, weakened therapeutic relationships, and limited integration of caring values into clinical decision-making [7–9].

Caring ability is not innate but gradually develops through supportive educational environments, caring practice, and reflective experience. Previous studies have shown that when nursing students learn in an environment rich in implicit care, they may internalize caring knowledge, imitate caring behaviors, and enhance their humanistic caring abilities [8, 17, 18]. Caring interactions among peers and between faculty and students are also positively associated with students’ humanistic caring competencies [19]. However, in China, although humanistic care education in nursing has received increasing attention, it remains insufficiently integrated into professional nursing courses. Many nursing programs still provide humanistic education mainly through theoretical instruction, with limited practical, experiential, and reflective components [12, 20, 21]. This separation makes it difficult for students to connect caring principles with clinical nursing practice, especially in pediatric nursing, where students need to integrate professional knowledge with empathy, ethical awareness, communication, and family-centered care.

Therefore, there is a need for a theory-informed pedagogical model that embeds humanistic caring education into pediatric nursing courses and evaluates its effects through both measurable outcomes and students’ reflective learning experiences. Tyler’s Model, also known as Tyler’s Objectives Model, provides a useful curriculum framework for aligning educational objectives, learning experiences, the organization of learning activities, and evaluation [22, 23]. Guided by this model, the “Humanities in Course” teaching model was developed to embed humanistic caring education into the pediatric nursing curriculum rather than deliver it as a separate humanities course. The model integrates caring knowledge, caring philosophy, caring behavior, and caring perception through theoretical teaching, experiential infant-care simulation, narrative reflection, situated learning, clinical practice, and caring atmosphere construction. Accordingly, this mixed-methods study aimed to evaluate the effectiveness of the “Humanities in Course” teaching model in improving nursing students’ humanistic caring abilities, caring climate, and learning satisfaction in pediatric nursing education.

### Research questions and hypotheses

This mixed-methods study was guided by the following research questions and hypotheses:

1. RQ1: Does the model improve students’ humanistic caring abilities, peer caring interaction, and perceived organizational caring climate?

H1–H3: Students’ scores on the Caring Ability Inventory, Peer Group Caring Interaction Scale, and Organizational Climate for Caring Questionnaire will be significantly higher after the course than before the course.

2. RQ2: How do students perceive the learning benefits of the model?

Descriptive expectation: Students will report positive perceptions in the Teaching Effectiveness Evaluation Questionnaire.

3. RQ3: How do students describe their development of humanistic caring after experiential learning activities?

Qualitative expectation: Narrative diaries will reflect perceived development in empathy, responsibility, ethical awareness, professional identity, communication, and child- and family-centered caring.

## Materials and methods

### Mixed methods design framework

This study adopted an equal-status concurrent mixed-methods design (QUAL + QUAN) to evaluate the “Humanities in Course” teaching model. This design was informed by the mixed-methods framework proposed by Schoonenboom and Johnson [24], which emphasizes the key dimensions of purpose, theoretical drive, timing, point of integration, planned versus emergent design, and design complexity. In this study, the quantitative and qualitative components were given equal priority because they addressed complementary aspects of the same research question: quantitative data assessed changes in caring ability, caring climate, and perceived teaching effectiveness, whereas qualitative narrative diaries explored students’ reflective experiences and internalization of humanistic caring.

**Fig. 1:**
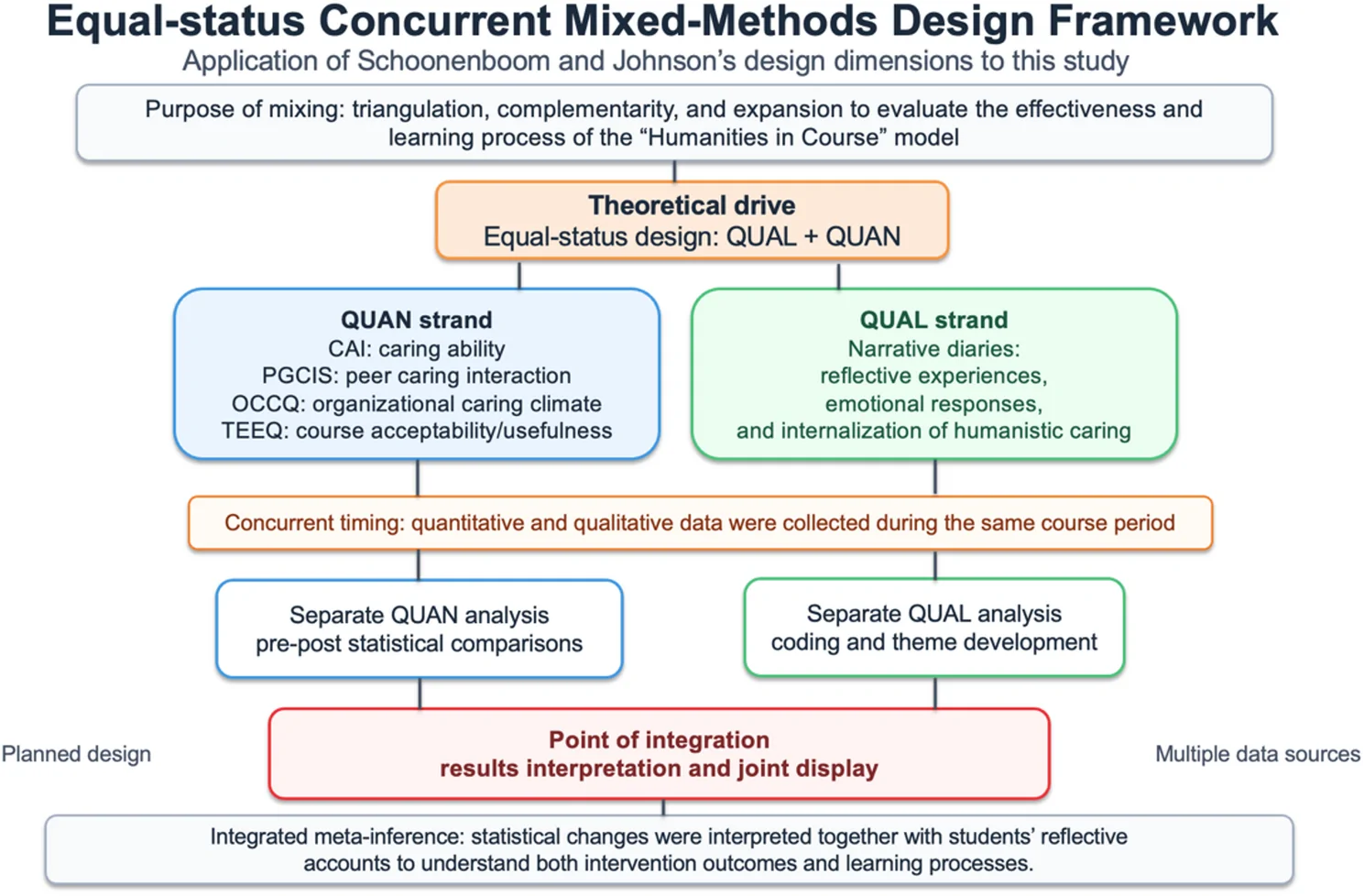
Mixed-methods research design framework

Quantitative data were collected using the CAI, PGCIS, OCCQ, and TEEQ, while qualitative data were obtained from students’ narrative diaries. The two components were implemented concurrently during the course period, analyzed separately, and integrated during the interpretation of findings. The purpose of integration was to achieve triangulation and complementarity by comparing statistical changes with students’ reflective accounts, thereby providing a more comprehensive understanding of the intervention effects. The overall mixed-methods design framework is shown in Fig. 1.

### Reporting framework

To enhance methodological rigor and transparent reporting, the manuscript was prepared in accordance with the Good Reporting of A Mixed Methods Study (GRAMMS) framework, which covers the rationale for mixed methods, design description, method-specific procedures, integration, methodological limitations, and insights generated through integration [25]. The completed GRAMMS checklist is provided in Supplementary File 1.

### Sampling and participants

A non-probability whole-population sampling method was employed in this study. The sample comprised all third-year undergraduate nursing students enrolled in the Pediatric Nursing course during the 2023 academic year. Inclusion was contingent upon provision of informed consent. Exclusion criteria included students with documented psychological disorders and those who took a leave of absence or withdrew from the course prior to its completion.

### Course design and implementation

The course aimed to enhance the humanistic caring abilities of nursing students. It was delivered over 11 weeks and consisted of 72 sessions in total, including 40 theoretical sessions and 32 practical sessions; each session lasted 45 min. Guided by the literature review, preliminary evidence regarding factors associated with caring ability, and the teaching objectives, the main teaching content was organized around four components: caring knowledge, caring philosophy, caring behavior, and caring perception [5, 6, 26, 27]. Experiential learning theory, narrative nursing theory, and situated learning theory served as the theoretical foundations for the course design [10, 28].

Accordingly, the teaching methods included experiential teaching using RealCare Baby^®^ 3, narrative teaching through narrative diaries, situational teaching through scenario simulation and role play, clinical practice, and the creation of a humanistic caring atmosphere.

**Fig. 2:**
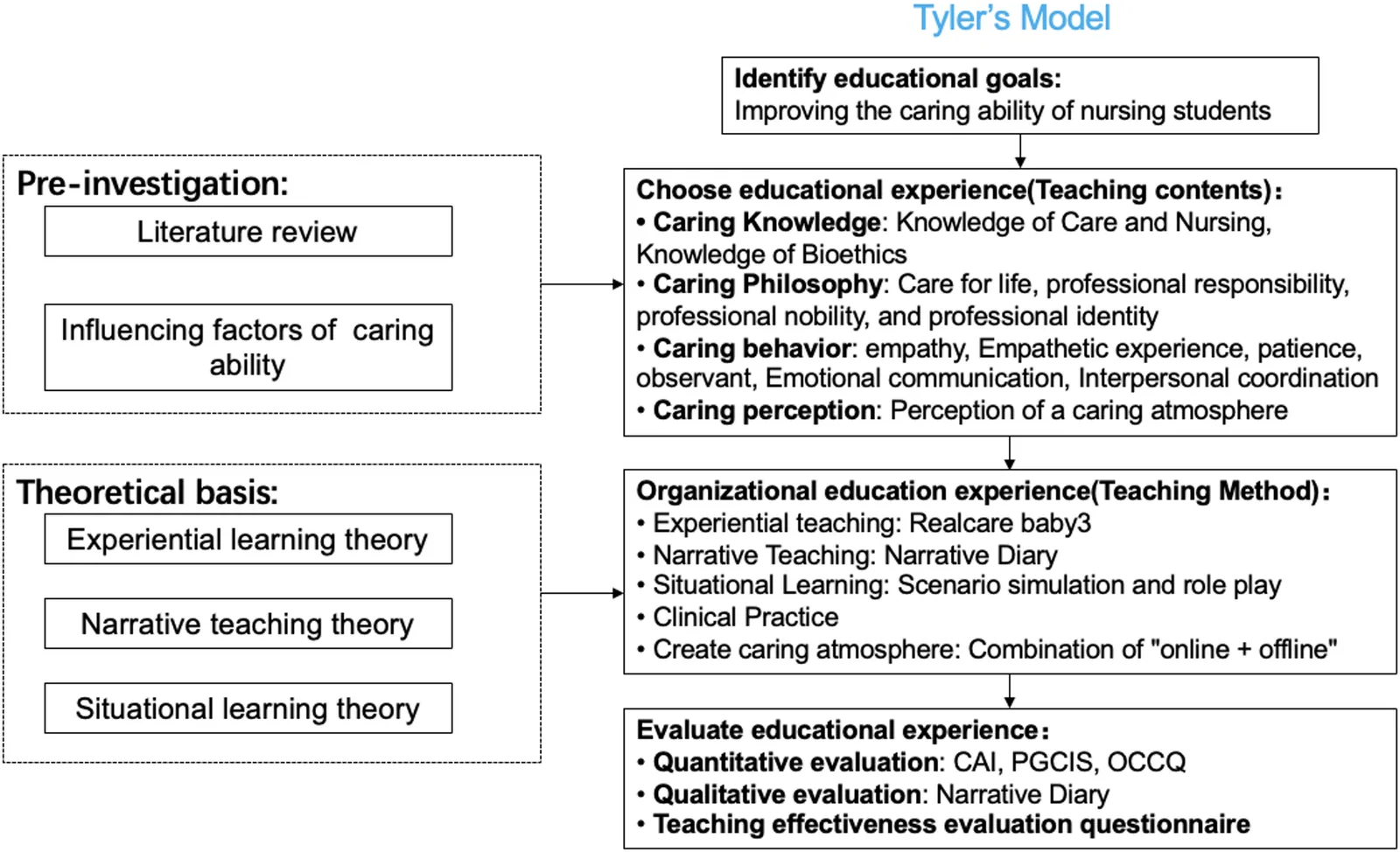
Diagram of the instructional design construction process

Tyler’s objectives model was used as the overarching framework to integrate the teaching objectives, teaching content, teaching methods, and evaluation strategy into a closed-loop instructional design, as shown in Fig. 2 [23, 29]. Within this framework, the change mechanism of the “Humanities in Course” model was conceptualized as a progression from knowledge and value input, to experiential learning and narrative reflection, to internalization of caring behaviors, formation of a caring climate among peers and between teachers and students, and improved course satisfaction and acceptability. Accordingly, the CAI, narrative diaries, PGCIS, OCCQ, and TEEQ were selected to evaluate these corresponding components of the model. Table 1 displays examples of the organization and structure of instructional materials used in the course.

**Table 1:** Instructional material organization and structure

Teaching Objectives	Teaching Content
Caring Knowledge	Nursing theory study: humanistic caring theory, etc. Observational learning in the labor ward: welcoming new life and experiencing bioethics.
Caring Philosophy	Humanities cases: nurses’ moral support for children with leukemia. Kindergarten practice: patterns and judgments of children’s growth and development.
Caring Behavior	Communication theory: discussing clinical situations. Clinical practice: small group reports on hospital visits. RealCare Baby^®^ 3: simulation of infant care.
Caring Perception	Create a caring atmosphere: teacher role models. WeChat public platform: nursing humanistic care case studies.

The course was delivered by a multidisciplinary teaching team of 13 members, including five theoretical instructors, seven clinical practice instructors, and one teaching assistant. The theoretical instructors were senior nursing faculty members with postgraduate training in nursing or related fields, and the clinical practice instructors had extensive experience in pediatric nursing. This team composition supported the integration of humanistic education, nursing theory, and pediatric clinical practice.

### Teaching method

The model used six integrated teaching strategies:

- *Theoretical teaching*: A Nursing Humanities Casebook was developed and integrated into the pediatric nursing curriculum, addressing humanistic, ethical, bioethical, and situational cases.
- *Experiential learning*: The RealCare Baby® 3 simulator was used to provide an immersive infant-care experience and to support responsibility, empathy, and problem-solving [11, 16, 30].
- *Narrative learning*: Students wrote reflective diaries within one week after the infant-care experience to promote self-reflection and internalization of caring experience [7, 31].
- *Situated learning*: Students designed and performed small-group scenario cases to analyze pediatric patients’ problems from professional and holistic perspectives and to develop empathy and emotional communication [10].
- *Clinical practice*: Practical clinical experiences were included in neonatal units, NICUs, and pediatric wards to connect classroom learning with pediatric nursing practice.
- *Caring atmosphere construction*: Humanistic stories, multimedia materials, teacher role modeling, and a WeChat public platform were used to enhance teacher-student interaction and cultivate a caring atmosphere [12, 32].

### Evaluation tools for teaching effectiveness

**Fig. 3:**
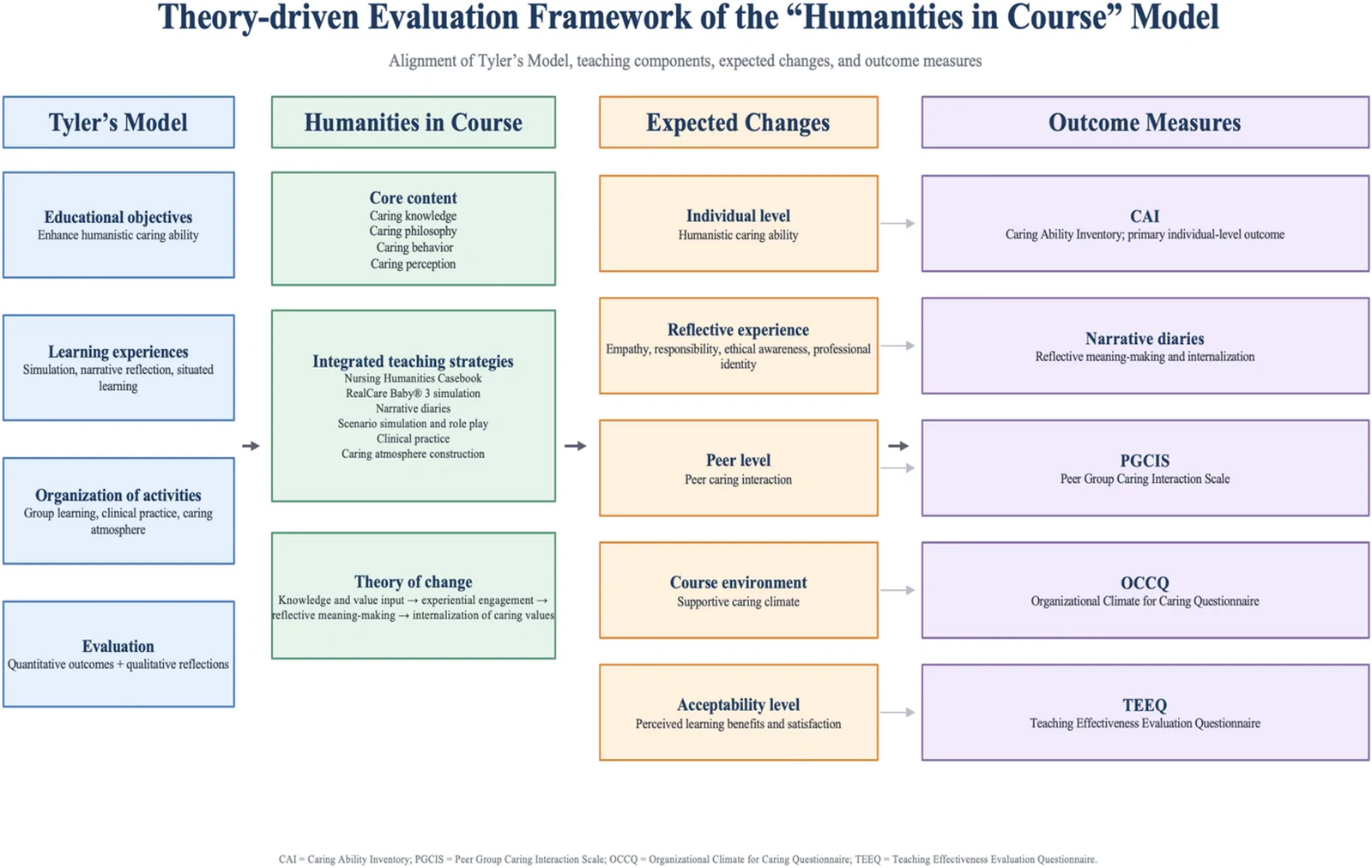
Theory-driven evaluation framework of the “Humanities in Course” model

The effectiveness of the “Humanities in Course” model was evaluated using quantitative questionnaires, narrative diaries, and a course evaluation questionnaire. The selection of outcome measures was guided by Tyler’s objectives model and the theory-driven evaluation framework shown in Fig. 3.

The Caring Ability Inventory (CAI) assessed students’ humanistic caring ability across Knowledge, Courage, and Patience [33, 34]. The Peer Group Caring Interaction Scale (PGCIS) assessed caring and supportive interactions among students [35, 36]. The Organizational Climate for Caring Questionnaire (OCCQ), based on Noddings’s caring framework, assessed students’ perceived caring climate in the educational setting; a revised Chinese version was used in this study [37, 38]. The self-developed Teaching Effectiveness Evaluation Questionnaire (TEEQ) assessed students’ perceived learning experience, satisfaction, and perceived usefulness of the course, with an English version provided as Supplementary File 2. Narrative diaries captured students’ reflective experiences after the RealCare Baby^®^ 3 activity.

### Data collection

After written informed consent was obtained, the researcher explained the study purpose, procedures, and requirements to the participants. Quantitative data were collected using paper-based questionnaires. The Caring Ability Inventory (CAI), Peer Group Caring Interaction Scale (PGCIS), and Organizational Climate for Caring Questionnaire (OCCQ) were administered within one week before the start of the course in March 2023 and again within one week after completion of the course in June 2023. The same instruments and standardized instructions were used at both time points. The Teaching Effectiveness Evaluation Questionnaire (TEEQ) was administered after the course to assess students’ perceptions of the “Humanities in Course” model.

Qualitative data were collected through students’ narrative diaries after the RealCare Baby^®^ 3 experiential learning activity. Students were required to write a reflective diary within one week after completing the infant-care simulation. The diary prompt encouraged students to describe the caregiving process, their emotions and experiences, what they learned from the activity, perceived changes in themselves, and reflections on pediatric nursing and humanistic caring. A total of 75 narrative diaries were collected.

Several procedures were used to reduce potential bias. Students were informed that participation was voluntary, that there were no right or wrong answers, and that their responses would be anonymized and used only for research purposes. Participation, non-participation, or withdrawal did not affect course grades or academic evaluation. All questionnaire data and narrative diaries were checked for completeness, anonymized, and prepared for analysis.

### Data analysis

Quantitative data were analyzed using SPSS 27.0. Continuous variables were described as mean ± standard deviation for normally distributed data or median and interquartile range for skewed data. Paired-sample t-tests were used to compare pre- and post-course scores on the CAI, PGCIS, and OCCQ. The total scores of the CAI, PGCIS, and OCCQ were treated as the primary quantitative outcomes, while subscale scores were treated as exploratory outcomes. To reduce the risk of type I error due to multiple subscale comparisons, Holm-Bonferroni correction was applied to exploratory subscale-level analyses. The TEEQ was analyzed descriptively using frequencies and percentages because it was administered only after the course to assess students’ perceptions of the teaching model. Statistical significance was set at α = 0.05.

Qualitative data from the narrative diaries were analyzed using Colaizzi’s seven-step method and managed with NVivo 12. All diaries were anonymized and assigned numerical labels before analysis. Two researchers independently read and coded the diaries, extracted significant statements, formulated meanings, and developed preliminary themes. Coding results were compared, and disagreements were resolved through consensus discussion with reference to the original text and input from the research team. An audit trail was maintained to document coding decisions, analytic memos, theme development, and revisions. Representative quotations were purposively selected according to their relevance, clarity, and ability to reflect students’ original meanings. Data adequacy was confirmed when no new major themes emerged after reviewing all 75 diaries. Formal participant checking was not conducted because the data were course-based reflective diaries collected after the learning activity; this was acknowledged as a limitation. Credibility, dependability, confirmability, and transferability were supported through independent coding, consensus discussion, audit trail maintenance, reflexive team discussion, and detailed description of the study context.

Integration occurred at the interpretation stage after quantitative and qualitative analyses were completed separately. Quantitative changes in CAI, PGCIS, OCCQ, and TEEQ results were compared with qualitative themes from the narrative diaries to examine convergence and complementarity. The integrated findings were summarized using a joint display.

### Ethical considerations

The principles of the Declaration of Helsinki were applied throughout the study in the following ways. First, respect for participants’ autonomy was ensured by providing all students with clear information about the study purpose, procedures, voluntary participation, and the right to withdraw at any time without penalty. Second, informed consent was obtained before data collection. Third, participant privacy and confidentiality were protected by anonymizing or coding questionnaire data and narrative diaries, removing identifying information, and restricting data access to the research team. Fourth, the study involved minimal risk because the intervention was embedded within a routine educational course and did not include invasive procedures or clinical treatment. Fifth, fairness and non-coercion were emphasized by ensuring that participation or non-participation did not affect students’ course grades, academic evaluation, or relationship with instructors. Finally, the study received prior approval from the Ethics Committee of School of Nursing Central South University [approval number: 2019054], and all procedures were conducted in accordance with approved ethical protocols.

### Generative AI disclosure

During manuscript preparation, generative artificial intelligence tools were used only for language polishing and grammar refinement. The tools were not used for data collection, data analysis, interpretation of results, figure generation, or decision-making. All AI-assisted outputs were critically reviewed, revised, and approved by the authors, who take full responsibility for the accuracy, integrity, and originality of the manuscript.

## Results

### Participants’ sample and characteristics

**Fig. 4:**
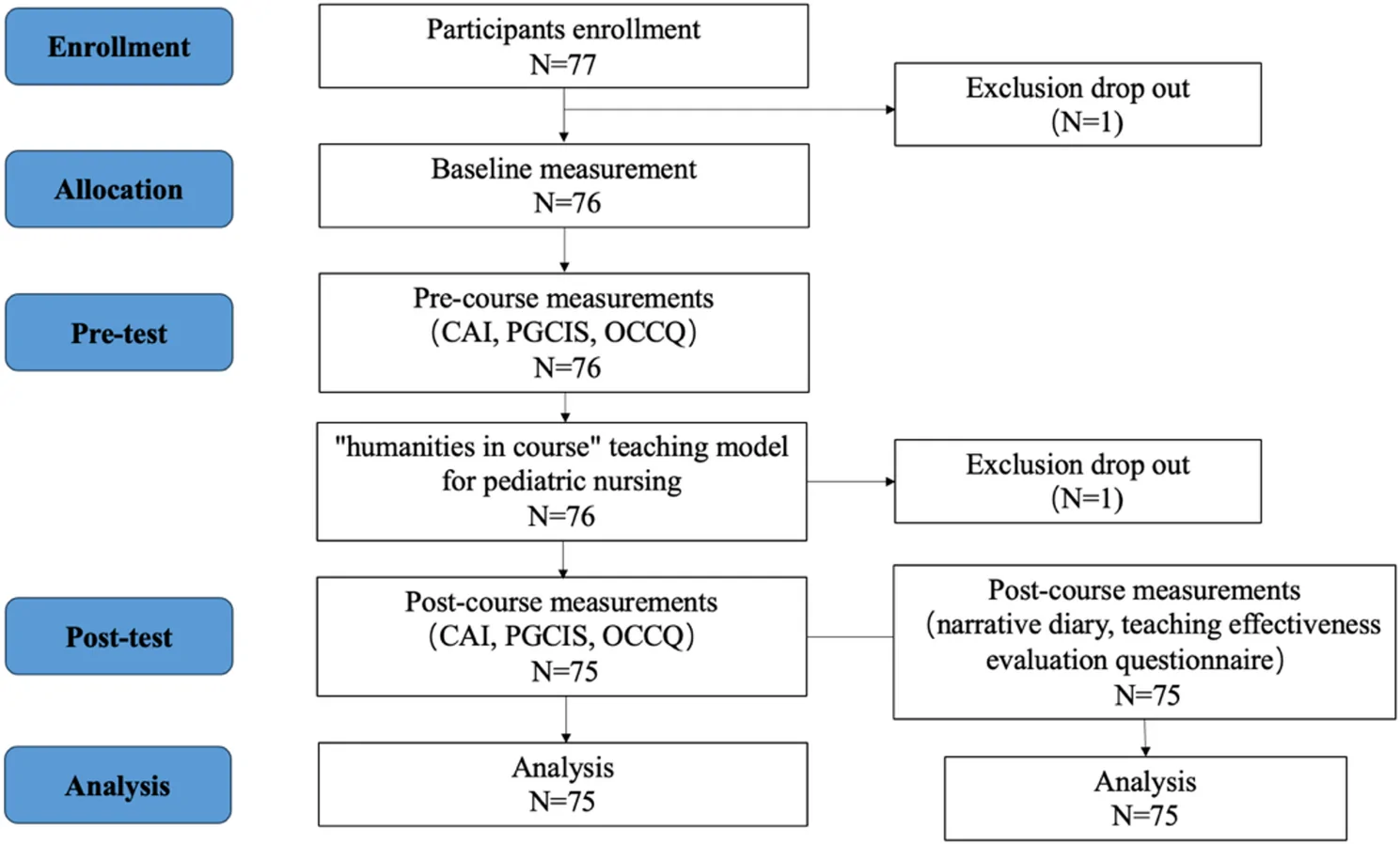
Flow diagram of participants in this study

A total of 77 students participated in the study. One student chose not to complete the pre-course humanistic care measure due to personal reasons, and another student took a leave of absence midway through the course. Although these two students were allowed to continue with the course for ethical reasons, their data were excluded from the analysis. Thus, the final analysis included data from 75 students who completed all course requirements and provided both pre- and post-course data. Additionally, narrative diaries from all 75 students were collected for content analysis, as illustrated in Fig. 4.

For pre-course measurements in March 2023, all 75 questionnaires were returned, achieving a 100% response rate. The same response rate was maintained for the post-course measurements in June 2023. The mean age of participants was 21.88 ± 0.71 years, with 64 (85.3%) identifying as female and 11 (14.7%) as male. Regarding their place of residence, 64.0% (*n* = 48) were from urban areas, while 36.0% (*n* = 27) were from rural regions. The reasons for choosing the nursing profession varied: 26.7% (*n* = 20) were self-motivated, 28.0% (*n* = 21) made the decision based on external advice, 16.0% (*n* = 12) transferred from other fields, and 29.3% (*n* = 22) cited other reasons. When asked about their level of enjoyment of the nursing profession, 52.0% (*n* = 39) expressed a positive attitude, including 5.3% (*n* = 4) who reported a strong liking, while 33.3% (*n* = 25) were uncertain, and 14.6% (*n* = 11) reported disliking the profession to varying degrees. Regarding their willingness to work in nursing after graduation, 53.4% (*n* = 40) indicated a willingness to pursue a nursing career, 30.7% (*n* = 23) were uncertain, and 16.0% (*n* = 12) were reluctant to enter the profession.

### Improvement of caring abilities

The CAI total score increased significantly from 188.11 ± 18.69 before the course to 203.57 ± 16.26 after the course (*p* < 0.001). In exploratory subscale analyses, Knowledge, Courage, and Patience scores increased after the course; however, after Holm-Bonferroni correction, only Knowledge and Courage remained statistically significant, as shown in Table 2.

**Table 2:** Pre- and post-course caring ability scores (mean ± SD)

Dimension	Pre-course	Post-course	t value	Raw *p* value	Holm-adjusted *p* value
CAI Total score	188.11 ± 18.69	203.57 ± 16.26	-5.674	< 0.001	—
Knowledge	74.43 ± 9.31	78.97 ± 6.81	-3.577	< 0.001	0.008
Courage	54.29 ± 11.35	63.53 ± 9.00	-6.005	< 0.001	0.008
Patience	59.39 ± 5.83	61.07 ± 3.92	-2.045	0.044	0.088

Raw *p* values were obtained from paired-sample t-tests. Holm-adjusted *+* values were calculated for exploratory subscale comparisons only. Total scores were treated as primary outcomes and were not included in the Holm-Bonferroni correction. For raw *p* values reported as < 0.001, 0.001 was used for correction

### Improvement of caring atmosphere

The total scores of the PGCIS and OCCQ increased significantly after the course. After Holm-Bonferroni correction, PGCIS Modeling and all OCCQ subscales remained statistically significant, whereas PGCIS Giving Assistance did not, as shown in Table 3.

**Table 3:** Comparison of pre- and post-course scores on peer group caring interaction and organizational climate scales (mean ± SD)

Dimension	Pre-course	Post-course	t value	Raw *p* value		Holm-adjusted *p* value
PGCIS Total score	70.08 ± 9.57	73.36 ± 7.64	-2.854	0.006	—	
Giving assistance	38.25 ± 5.78	39.73 ± 4.70	-2.017	0.047	0.088	
Modeling	31.83 ± 4.90	33.63 ± 3.83	-3.162	0.002	0.008	
OCCQ Total score	139.65 ± 18.30	149.33 ± 13.45	-5.014	< 0.001	—	
Modeling	63.88 ± 8.81	69.08 ± 7.13	-5.312	< 0.001	0.008	
Dialogue	42.63 ± 6.26	44.89 ± 4.79	-3.187	0.002	0.008	
Confirmation	33.15 ± 4.20	35.36 ± 3.53	-4.454	< 0.001	0.008	

Raw *p* values were obtained from paired-sample t-tests. Holm-adjusted *p* values were calculated for exploratory subscale comparisons only. Total scores were treated as primary outcomes and were not included in the Holm-Bonferroni correction. For raw *p* values reported as < 0.001, 0.001 was used for correction

### Analysis of the content of narrative diaries

After the course, 75 students submitted narrative diaries, resulting in the identification of five key themes: “gratitude to parents,” “professional quality development,” “bioethics,” “operational learning,” and “course experience.” These themes reflect the development of humanistic caring qualities, including caring concepts, caring knowledge, and caring behaviors. The effectiveness and methodology of the course were also acknowledged by the students. Detailed findings are presented in Table 4.

**Table 4:** Results of themes analysis of narrative diaries

Themes	Subthemes	Examples of original text
Gratitude to parents	Great Parents Understanding parental devotion Feeling the difficulty of parenthood	“It takes a lot of love and sacrifice to feed your children.”; “ My strongest sensation and experience is still the hard effort and difficulties of parenting.”; “Parenting is more than just a few words, you must experience it to fully comprehend it.”; “It makes me feel how much my mom and dad have done for me and how great they are.”
Professional quality development	Sense of responsibility Empathy Passion for the profession Love and humanistic caring Patience carefulness	“I’ve gained so much and love the nursing profession even more”; “It also caused me to realize how important some of the details of clinical work are, and how mistakes are more likely to occur if you are not careful.”; “These few days of invaluable experience will also help me to be less flustered at work in the future, and to be more calm, careful, and patient when caring for children.”; “In nursing work, we need to think empathetically about the needs of the people we care for and stay close to their point of view.”; “I hope to keep this pure love and incorporate it into my work, turning it into a passion to do something that can truly benefit patients.”; “We must have good professional and scientific qualities to effectively solve children’s health problems, as well as good physical and moral qualities, respect for children, loyalty and dedication to children’s health.”; “In just 48 h, I have not only mastered the knowledge and techniques from theory to practice, but I have also become more caring and patient with children.”; “Therefore, as nurses, we need to understand the anxiety of the parents and other family members to a greater extent. In our work, we not only need to take care of the child’s mood, but also take care of the family’s mood, to maximize to ease their worries.”
Bioethics	Reverence for life Caring for Life The power of new life Vulnerability of young lives Responsibility for every life	“The students around us were all full of tears, a shock of new life”; “Every new life is a cause for celebration. We were all young once, and they have characteristics and needs that are different from those of adults, as well as relatively more vulnerable.”; “All this makes me feel the weight of life and the duty to guard it.”; “No matter what stage of life they are in, no matter whether they have sufficient knowledge of the world or not, they all need love and care.”
Operational learning	Daily care of infants The right way to care	“For the first time, I was able to put my theoretical knowledge of pediatric nursing into practice, and for the first time, I was able to perform practical exercises with repetition and feedback.”; “I began to learn to place the items needed to simulate an infant in a fixed position, to remember the importance of protecting the head and neck, and to learn not only to acquire the knowledge of caring for an infant on my own, but also to teach this correct knowledge to my own family.”; “I’ve learned a lot about baby care.”
Course experience feeling	Full of innovation A rewarding course	“The pediatric teaching faculty suggested an innovative method of instruction.”; “Thanks to this precious experience, I was able to gain a wealth that other people my age might not have been able to obtain, not only in theory, but also in spirit.”

### Teaching effectiveness evaluation survey

At the end of the course, students completed the Teaching Effectiveness Evaluation Questionnaire to assess their perceptions of the “Humanities in Course” model. Overall, students reported positive perceptions of the course. Most students agreed that the model was attractive (94.7%), enhanced their learning experience (92.0%), stimulated interest in the subject (89.3%), and helped them better understand theoretical concepts (93.3%). Students also reported perceived improvements in practical and professional abilities, including operational learning (92.0%), analytical problem-solving ability (96.0%), communication with patients and families (94.7%), patience when interacting with patients (98.7%), expression of concern for patients (93.3%), proactive understanding of patients’ needs (96.0%), and providing reassurance and comfort in future clinical practice (97.3%).

## Discussion

This mixed-methods study evaluated the “Humanities in Course” model as an approach to embedding humanistic education into pediatric nursing education. The quantitative findings showed significant improvements in the total scores of the CAI, PGCIS, and OCCQ after the course, suggesting positive changes in students’ overall humanistic caring ability, peer caring interaction, and perceived caring climate. Qualitative findings further indicated that students perceived gains in gratitude to parents, professional quality development, bioethical awareness, operational learning, and course experience. Together, these findings suggest that the model may help connect humanistic caring principles with pediatric nursing knowledge, experiential learning, and reflective practice.

### Interpretation of findings

The increase in the CAI total score suggests that the model may support the development of students’ humanistic caring ability. This finding is consistent with the view that caring ability can be cultivated through education, caring practice, reflective experience, and supportive learning environments [33, 34, 39]. It also aligns with studies showing that humanistic education, narrative reflection, and experiential learning can promote empathy, professional development, and caring competence among nursing students [1, 7, 40].

The qualitative themes help explain how these changes may have occurred. The RealCare Baby^®^ 3 activity provided students with an experiential caregiving context, while narrative diaries encouraged reflection on responsibility, parental perspectives, ethical awareness, and child-centered care. These findings are consistent with evidence that experiential learning, narrative education, and pediatric simulation can support students’ engagement, empathy, and readiness for clinical practice [11, 16, 28, 31]. After Holm-Bonferroni correction, improvements in CAI Knowledge and Courage remained statistically significant, whereas CAI Patience did not. This suggests that knowledge and willingness to engage in caring relationships may be more responsive to short-term educational activities, while patience may require longer-term clinical exposure and repeated interactions with children and families.

The model also appeared to strengthen students’ perceptions of caring interaction and caring climate. The increases in PGCIS and OCCQ total scores may be related to group learning, scenario simulation, clinical practice, teacher role modeling, and caring atmosphere construction. Previous studies have suggested that peer interaction, teacher modeling, and caring educational environments contribute to nursing students’ caring attitudes and professional socialization [19, 35–37]. After Holm-Bonferroni correction, PGCIS Modeling remained statistically significant, whereas PGCIS Giving Assistance did not, suggesting that awareness of peer role modeling may have changed more readily than direct helping behaviors. For the OCCQ, Modeling, Dialogue, and Confirmation remained significant after correction, consistent with Noddings’s emphasis on modeling, dialogue, practice, and confirmation in caring education [32, 41].

The qualitative findings also indicated that students developed bioethical awareness and professional qualities, including empathy, responsibility, patience, carefulness, and concern for families. These findings suggest that students began to view pediatric nursing as both a technical and humanistic practice. This is consistent with studies emphasizing the importance of bioethics education, empathy, and professional identity formation in nursing education [7, 9, 42, 43]. Students also reported positive perceptions of the course in the TEEQ, suggesting good acceptability and perceived learning value. However, because the TEEQ was a post-course self-reported evaluation, these findings should be interpreted as descriptive evidence of students’ perceived learning experience rather than objective evidence of effectiveness.

### Strengths

This study has several strengths. First, it developed a theory-informed model that embedded humanistic caring education into a professional pediatric nursing course rather than delivering it as an isolated humanities course. Second, the course design and evaluation strategy were guided by Tyler’s objectives model and the theory of change of the “Humanities in Course” model, linking educational objectives, teaching strategies, expected changes, and outcome measures. Third, the mixed-methods design allowed quantitative changes to be interpreted together with students’ reflective experiences, providing a more comprehensive understanding of both outcomes and learning processes. Fourth, the model was evaluated from multiple perspectives, including individual caring ability, peer caring interaction, organizational caring climate, narrative reflection, and perceived course acceptability. Finally, the use of experiential simulation, narrative reflection, situated learning, clinical practice, and caring atmosphere construction may enhance the model’s practical relevance and transferability to similar pediatric nursing education contexts.

### Limitations

Several limitations should be acknowledged. First, the single-group pre–post design limits causal inference, as maturation, history, testing effects, and the Hawthorne effect could not be fully excluded. Second, all quantitative outcomes were self-reported, and the course-based narrative diaries may also have been affected by social desirability bias, although anonymity and separation from course grading were emphasized. Third, after Holm-Bonferroni correction, CAI Patience and PGCIS Giving Assistance did not remain statistically significant; therefore, subscale-level findings should be interpreted cautiously. Finally, this study was conducted in one educational context with a relatively small sample and did not include objective assessments of caring behavior, such as OSCE-based performance evaluation, teacher ratings, direct observation, or feedback from children and families.

### Future directions

Future studies should use controlled, multi-center, and longitudinal designs to evaluate the effectiveness and sustainability of the “Humanities in Course” model. A randomized or quasi-experimental design with a comparable control group would help strengthen causal inference. Follow-up assessments during or after clinical placement would help determine whether improvements in humanistic caring ability are sustained and transferred into practice. Future research should also include more objective and multi-source outcome measures, such as teacher ratings, peer assessment, OSCE-based assessment, observed clinical behaviors, reflective writing assessed by blinded reviewers, and feedback from children or families where appropriate. Further studies may explore which components of the model, such as infant-care simulation, narrative reflection, role play, clinical practice, or caring atmosphere construction, contribute most strongly to students’ development of humanistic caring competence.

## Conclusion

This study suggests that the “Humanities in Course” teaching model may be a feasible approach for embedding humanistic education into pediatric nursing education. The model was associated with improvements in students’ overall humanistic caring ability, peer caring interaction, and perceived caring climate, while qualitative findings indicated perceived development in professional quality, bioethical awareness, operational learning, and reflective understanding of pediatric caring. Students also reported positive perceptions of the course. These findings suggest that integrating experiential learning, narrative reflection, situated learning, and caring atmosphere construction may help connect humanistic caring values with pediatric nursing knowledge and practice. Future controlled, multi-center, and longitudinal studies with objective and multi-source outcome measures are needed to confirm the effectiveness, sustainability, and transferability of this model.

## Supplementary Information

Supplementary Material 1.

Supplementary Material 2.

## Data Availability

The datasets generated and/or analyzed during the current study are available from the corresponding author on reasonable request.

## Abbreviations

CAI: Caring Ability Inventory
PGCIS: Peer Group Caring Interaction Scale
OCCQ: Organizational Climate for Caring Questionnaire
TEEQ: Teaching Effectiveness Evaluation Questionnaire
MMR: Mixed Methods Research
