# Supplementary material for: A mixed-methods study of a “Humanities in Course” model: improving caring abilities, climate, and satisfaction in pediatric nursing education
Source: BMC Med Educ. 2026 Jun 29;26:1467. doi: 10.1186/s12909-026-09809-0 (PMC13576118; doi:10.1186/s12909-026-09809-0)
Supplement: Supplementary file 1 [file 12909_2026_9809_MOESM1_ESM.docx]

**Supplementary File 1. GRAMMS Checklist**

Manuscript title: A Mixed-Methods Study of a "Humanities in Course" Model: Improving Caring Abilities, Climate, and Satisfaction in Pediatric Nursing Education

Reporting guidance: Good Reporting of A Mixed Methods Study (GRAMMS) guidance (O'Cathain et al., 2008)

Purpose of this checklist: This supplementary file summarizes how the manuscript addresses the six GRAMMS reporting items for mixed-methods studies.

| **GRAMMS item** | **Where addressed in the manuscript** | **How the item was addressed in this study** |
| --- | --- | --- |
| **1. Describe the justification for using a mixed-methods approach to the research question** | Abstract; Background; Materials and Methods - Mixed Methods Design Framework | The study aimed to evaluate the effectiveness of the "Humanities in Course" teaching model in pediatric nursing education. A mixed-methods approach was used because quantitative data alone could show whether students’ caring ability, peer caring interaction, organizational caring climate, and course perceptions changed after the course, but could not fully explain how students experienced and internalized humanistic caring values. Qualitative narrative diaries provided contextual explanations of students’ reflective experiences after the RealCare Baby® 3 activity, including empathy, responsibility, bioethics, operational learning, and course experience. The two strands were therefore combined for complementarity and triangulation. |
| **2. Describe the design in terms of the purpose, priority, and sequence of methods** | Materials and Methods - Mixed Methods Design Framework; Figure 1 | The manuscript describes the study as a concurrent mixed-methods design with equal status given to the qualitative and quantitative components. Quantitative data were collected before and after the course, while qualitative narrative diaries were collected after the RealCare Baby® 3 experiential learning activity. The quantitative strand included pre- and post-course assessments using the Caring Ability Inventory, Peer Group Caring Interaction Scale, and Organizational Climate for Caring Questionnaire, as well as a post-course Teaching Effectiveness Evaluation Questionnaire. The qualitative strand consisted of students’ narrative diaries written after the RealCare Baby® 3 experiential learning activity. The two strands were analyzed separately and then integrated during interpretation. |
| **3. Describe each method in terms of sampling, data collection, and analysis** | Materials and Methods - Sampling and Participants; Data Collection; Data Analysis | A non-probability whole-population sampling method was used to recruit eligible third-year undergraduate nursing students enrolled in the Pediatric Nursing course. Seventy-five students who completed the course and provided both pre- and post-course data were included in the final analysis. Quantitative data were collected before and after the course using the CAI, PGCIS, and OCCQ, and students’ post-course perceptions were assessed using the TEEQ. Quantitative data were analyzed using descriptive statistics and paired-sample t-tests, with Holm-Bonferroni correction applied to exploratory subscale-level analyses. Qualitative data were collected through narrative diaries written within one week after the RealCare Baby® 3 experiential learning activity. The diaries asked students to reflect on the caregiving process, emotions and experiences, learning gains, perceived personal changes, pediatric nursing, and humanistic care. The diaries were anonymized, assigned numerical labels, managed using NVivo 12, and analyzed using Colaizzi’s seven-step method. |
| **4. Describe where integration occurred, how integration occurred, and who participated in integration** | Materials and Methods - Mixed Methods Design Framework; Data Analysis; Results; Discussion | Integration occurred primarily at the interpretation stage after separate quantitative and qualitative analyses. Quantitative findings were compared with qualitative themes to identify convergence and complementarity. For example, increased CAI scores were interpreted together with qualitative themes such as professional quality development, empathy, patience, responsibility, and bioethics. Improvements in PGCIS and OCCQ scores were interpreted alongside students’ descriptions of peer collaboration, teacher role modeling, and a supportive caring atmosphere. Positive TEEQ results were interpreted with diary themes related to operational learning and course experience. The research team jointly discussed quantitative and qualitative findings to develop integrated interpretations. The integrated interpretation was presented in the Data Analysis and Discussion sections to make this integration explicit. |
| **5. Describe any limitation of one method associated with the presence of the other method** | Discussion - Limitations; Future Directions | The manuscript acknowledges limitations associated with each strand and with their combination. The quantitative strand relied mainly on students’ self-reported outcomes, which may have been affected by social desirability bias, testing effects, maturation, and Hawthorne effects. The qualitative strand used course-based narrative diaries, which may also have encouraged socially desirable reflections because students wrote them in an educational context. Although integration of the two strands helped contextualize the quantitative findings, both strands were derived from students’ self-reports, and the study did not include external observation, teacher ratings, OSCE-based assessment, clinical performance assessment, or feedback from children and families. In addition, because the study used a single-group pre–post design without a control group, causal inference remains limited. |
| **6. Describe any insights gained from mixing or integrating methods** | Materials and Methods - Data Analysis; Results; Discussion - Interpretation of Findings | The integration of methods generated insights that could not be obtained from either strand alone. Quantitative results demonstrated measurable improvements in students’ caring ability and caring climate, while qualitative diaries explained how the intervention may have worked. The qualitative themes suggested that experiential infant-care simulation, narrative reflection, ethical awareness, empathy development, peer collaboration, and teacher role modeling helped students internalize humanistic caring values. Together, the integrated findings suggest that the "Humanities in Course" model may improve humanistic caring ability not only by increasing knowledge, but also by creating embodied, reflective, and emotionally meaningful learning experiences within a supportive caring climate. |

**Reference**

O'Cathain A, Murphy E, Nicholl J. The quality of mixed methods studies in health services research. Journal of Health Services Research & Policy. 2008;13(2):92-98. doi:10.1258/jhsrp.2007.007074.
