# Supplementary material for: A mixed-methods study of a “Humanities in Course” model: improving caring abilities, climate, and satisfaction in pediatric nursing education
Source: BMC Med Educ. 2026 Jun 29;26:1467. doi: 10.1186/s12909-026-09809-0 (PMC13576118; doi:10.1186/s12909-026-09809-0)
Supplement: Supplementary file 2 [file 12909_2026_9809_MOESM2_ESM.docx]

**A teaching effectiveness evaluation questionnaire (TEEQ)**

Response options (5-point Likert scale):
1 = Strongly agree	2 = Agree	3 = Neutral	4 = Disagree	5 = Strongly disagree

| No. | Item | Strongly agree | Agree | Neutral | Disagree | Strongly disagree |
| --- | --- | --- | --- | --- | --- | --- |
| 1 | I find the experiential teaching sessions appealing. |  |  |  |  |  |
| 2 | This experiential teaching can improve my learning outcomes. |  |  |  |  |  |
| 3 | It can stimulate my interest in this subject. |  |  |  |  |  |
| 4 | It can help me better understand and master theoretical knowledge. |  |  |  |  |  |
| 5 | It can foster my logical thinking ability. |  |  |  |  |  |
| 6 | It can deepen my understanding and mastery of practical skills/operations. |  |  |  |  |  |
| 7 | It can improve my ability to analyze and solve problems. |  |  |  |  |  |
| 8 | It can improve my hands-on/practical skills. |  |  |  |  |  |
| 9 | It can enhance my ability to adapt to changing situations. |  |  |  |  |  |
| 10 | It helps to bridge the gap between school and clinical practice. |  |  |  |  |  |
| 11 | It helps improve my communication with patients and their family members. |  |  |  |  |  |
| 12 | It helps me understand patients’ emotions. |  |  |  |  |  |
| 13 | It helps me put myself in patients’ shoes and understand and identify with their situation. |  |  |  |  |  |
| 14 | It helps me increase my patience with patients. |  |  |  |  |  |
| 15 | It helps me express care for patients appropriately. |  |  |  |  |  |
| 16 | It helps me accompany patients as much as possible during internship or work. |  |  |  |  |  |
| 17 | It helps me take the initiative to observe patients during internship or work. |  |  |  |  |  |
| 18 | It helps me take the initiative to understand patients’ needs during future internship or work. |  |  |  |  |  |
| 19 | In future internship or work, when patients are emotionally unstable, I will provide more comfort and reassurance. |  |  |  |  |  |
| 20 | It helps me understand my parents and feel grateful to them. |  |  |  |  |  |

21. Other comments or suggestions about this teaching method:

________________________________________________________________________________

________________________________________________________________________________

________________________________________________________________________________
